# Occupational heat strain among home health care workers: a cross-sectional study

**DOI:** 10.1186/s12889-026-28969-8

**Published:** 2026-09-29

**Authors:** Remo Fortunato, Martina S. Ragettli, Tiziano Gerosa, Julia Dratva, Isabel Baumann

**Affiliations:** 1 Department of Health Sciences, Institute of Public Health, Zurich University of Applied Science, Katharina-Sulzer-Platz 9, Winterthur, 8400 Switzerland; 2 Swiss Tropical and Public Health Institute, Allschwil, Switzerland; 3 University of Basel, Basel, Switzerland; 4 University of Applied Sciences and Arts of Southern Switzerland, Manno, Switzerland

**Keywords:** Heat strain, Home care workers, Home health care workers, Survey, Climate change, Switzerland, Occupational health, Prevention, Public health

## Abstract

**Background:**

Extreme heat events affect workers’ health. Home health care workers face physically strenuous tasks, mobility demands, and increased workloads during heat events. Yet little is known about the extent of heat strain and the specific symptoms they experience. This study aims to examine how home health care workers in Switzerland perceive heat exposure at work and what heat-related symptoms they experience. We examine which factors are associated with perceived heat strain.

**Methods:**

We conducted an anonymous online cross‑sectional survey of home health care workers (*N* = 359) in north-eastern and Southern Switzerland, two climatically distinct regions. Using items from previous studies on occupational heat strain, we assessed perceived heat strain, heat-related symptoms and a set of independent variables. Differences were tested using Mann–Whitney U and chi‑square tests, followed by ordinal logistic regression to identify associations and regional moderation effects.

**Results:**

Substantial perceived heat strain was reported by 86% of participants. Higher levels of perceived heat strain were significantly associated with increased workload (OR 14.06, 95% CI 7.12–28.85), heat-related symptoms (OR 5.27, 2.20–12.88), constrained hydration (OR 2.59, 1.35–5.02), working in southern Switzerland (OR 3.98, 2.11–7.61), and seeking cool environments to rest (OR 3.78, 1.55–9.60).

**Conclusions:**

Home health care workers reported substantial perceived heat strain at work, particularly when workload increased or hydration was constrained. Regional differences were evident, with stronger heat strain in southern Switzerland. Findings highlight the need to develop heat-protection measures tailored to workplace characteristics—such as workload, constrained hydration, and geographical region—to strengthen climate resilience in this essential and understudied workforce.

**Supplementary Information:**

The online version contains supplementary material available at https://doi.org/10.1186/s12889-026-28969-8.

## Background

Extreme heat events constitute a substantial risk for human health [1–6]. While elderly people and infants are particularly vulnerable, the working population may also face substantial risks. Specifically, occupational groups who spend time outdoors or perform physically demanding tasks are at increased risk of heat-related health effects [7–9]. At work, negative effects of heat stress may occur even at temperatures lower than those for which public warnings are issued [10, 11]. This is, for instance, due to physical activity increasing endogenous heat production, direct sun exposure, or the need to wear personal protective equipment (PPE), which hinders heat dissipation [9]. 

Although a large body of research examined heat stress in outdoor workers [7, 9, 12–15], there have been comparatively few studies regarding the impact of heat on health care workers. Those that examined health care workers mostly focused on the use of personal protective equipment (PPE) on heat strain during the COVID-19 pandemic [16]. Health care workers exposed to heat stress—primarily while wearing PPE—frequently reported some combination of the following symptoms: headaches; excessive or profuse sweating; thirst; fatigue or exhaustion; impaired concentration; and dizziness, light‑headedness, or faintness [16, 17]. A systematic review of the literature on heat-related symptoms shows that exertional heat illnesses and exertional heat stroke were positively associated with heat exposure [18]. Furthermore, in a controlled experiment using an environmental chamber, Quartucci et al. (2024) found that exposure to heat had a negative effect on thermal, physical, and psychological stress. That is, heat-induced discomfort (assessed via a visual analogue scale); perceived exertion and effort during health care tasks (measured using the NASA Task Load Index and Likert-scale items); and mental strain such as concentration difficulties, or nervousness (evaluated through self-reported scores and adapted health care workers survey items) [19]. A survey from the United Kingdom found that 91.5% of health care workers perceived occupational heat stress to impair their cognitive performance, and 20% reported heat-related absenteeism [14]. 

Home health care workers, as a mobile subset of health care workers, commute between clients’ residences, often under time constraints, and perform physically strenuous tasks such as lifting people, contributing to both physical strain and heat exposure. Additionally, their workload increases during heat episodes, as many of their clients are vulnerable to heat-related health effects [20]. In 2024, home care organizations in Switzerland provided support to 476’413 clients. Health care and support activities were carried out by 68’707 workers. In this study, we focus on those workers who are active in health care which is the majority of the workers. In 2024, these workers provided a total of 25,637,002 h of health care as compared to the workers in housekeeping and social care services who provided a total of 6,020,728 h [21, 22]. 

Heat-related health risks among home health care workers may be mitigated by climate change adaptation measures tailored to the needs and circumstances of this largely under-researched population [4]. To the authors' knowledge at the time of writing, no studies have specifically examined the extent to which health and well-being of home health care workers is affected by heat strain at work. Protecting home health care workers from heat-related health impacts is important amidst a warming climate and an ageing population increasing the need for care.

To address this gap, we conducted a survey among home health care workers in Switzerland to gain a better understanding of the prevalence of perceived heat exposure among home health care workers at work and to examine heat-related behaviour in the study population, for the purpose of developing preventive measures tailored to home health care workers’ needs subsequently. The survey was conducted in north-eastern Switzerland and southern Switzerland to capture two climatically different regions.

## Methods

### Study design

We conducted an anonymous cross-sectional online survey among a convenience sample of home health care workers in north-eastern and southern Switzerland which ran from late August until late September 2024. North-eastern Switzerland (cantons of Zürich and Thurgau, German-speaking) has a temperate climate with warm summers and cold winters (mean temperatures: January 2024, 1.4 °C; August 2024, 20.4 °C [23]). In contrast, southern Switzerland (canton of Ticino, Italian-speaking) features a Mediterranean climate characterized by milder winters, and higher solar insolation. It is the warmest region in Switzerland (mean temperatures: January 2024, 5.2 °C; August 2024, 24.8 °C [24, 25]). In the weeks before and during the data collection, temperatures repeatedly rose above 30 °C. For example, the city of Zurich, located in north-eastern Switzerland, recorded temperatures above 30 °C on ten days in August [26]. 

In Switzerland, home health care services (locally referred to as Spitex) constitute a highly regulated and formal component of the national health care infrastructure, providing professional nursing and ambulatory care within patients’ private residences. These services are delivered by a range of professionals—comprising specialized nurses, nursing assistants, and certified health care workers—as part of a structured, organized system of community-based outpatient care, distinct from informal or domestic caregiving.

In north-eastern Switzerland the recruitment took place in two large home health care organizations; In southern Switzerland, 193 freelance home health care workers form a public list, 20 associations, 38 private and two public home health care organizations were contacted. We sent the link to the online survey to team leaders in home health care organizations and to publicly available lists of freelance home health care workers and related associations, inviting them to participate anonymously and to share the link with their colleagues (i.e., snowball sampling). The program Redcap [27, 28] was used for dissemination and data collection.

The online questionnaire assessed occupational heat exposure and associated health outcomes, including self-reported history of heat-related symptoms during work, perceived heat strain, and the use of individual preventive measures, as well as employer-provided prevention measures. To capture heat exposure during work, respondents were asked about their primary mode of transportation, changes in hydration behaviour (e.g., restricted water intake), and perceived workload changes on hot days.

The instrument was informed by validated questionnaires from prior occupational heat stress studies [29–31], ensuring content relevance and methodological consistency. The central item of the present study, “perceived heat strain” was assessed via a single survey item identical to the one used in a Swiss study by Martucci and colleagues to accommodate the time constraints of mobile care work [30]. While subjective perception does not directly equate to individual physiological strain, O’Connor et al. demonstrated that self-reported heat strain maintains a significant linear relationship with objectively measured physiological indices across moderate occupational workloads, supporting its utility as a pragmatic, population-level indicator of environmental heat exposure [32]. The item on workload change was used based on a study from the United Kingdom by Williams et al. [31] The item on break in cool areas and constrained hydration was based on a German study by Jegodka et al. [29] The questionnaire was developed with the support from collaborators with a background in nursing who also tested the questionnaire in both languages, to avoid language-based measurement errors. Electronic consent was obtained upon initiation of the survey. An English translation of the Questionnaire can be found under supplementary material, or on the SWISSUbase repository together with the data set https://doi.org/10.48573/nrhm-sg66.

### Outcome and covariates

The main outcome variable, heat strain, was assessed by asking, “How strenuous is the heat for you at work on hot days (> 30°C)?” Response levels were, (i) not at all, (ii) a little strenuous, (iii) rather strenuous, and (iv) very strenuous. Since merely 3 people reported (i) no heat strain at all, we merged them with (ii) little heat strain for further analysis.

Covariates are sex (male, female), age (in years), education (basic training, apprenticeship, college of higher education, university, other), region (southern Switzerland, north-eastern Switzerland), smoking (yes, no, no answer), pre-existing medical conditions (yes, no).

Independent variables are workload change on hot days (> 30 °C) (increased a lot, increased somewhat, same, decreased somewhat, decreased a lot), heat-related symptoms (yes, no), heat adaptation measures (nothing, avoid exertion, reduce exertion, seek cool environment, adjust clothing, cool body, hydrate, light eating, other), means of transportation (bike, e-Bike, car, public transport, other), break in cool area (yes, no, sometimes), constrained hydration (yes, no, sometimes).

### Statistical analysis

To assess whether the distribution of responses differed between the two regions, Mann-Whitney-U, respectively, chi-square tests were conducted for the outcome variable and each variable of interest.

Thereafter, an ordinal logistic regression model was estimated to explore the effect of the variables of interest on the outcome of perceived heat strain. Additionally, we tested an ordinal logistic regression model, including region as an interaction term, to evaluate potential moderating effects of regional differences on “workload change on hot days”, “break in cool areas”, and “constrained hydration”. These interactions were not significant and failed to improve model fit, thus they were discarded. The results of the regression analysis are reported as odds ratios (OR). All data was analysed using R (version 4.5.1) and R Studio (2025.5.1.513) [33, 34]. Ordinal logistic regression models were calculated using polr() from the package MASS [35]. 

## Results

### Descriptive statistics

We reached a sample of 359 participants, of whom 62% were from southern Switzerland and 38% were from north-eastern Switzerland. The mean age was 44.8 years (range: 18–66) and 88% of the respondents were female. The distribution of socio-demographic variables is reported in Table 1.

**Table 1:** Distribution of covariates

Study population, count (%)		359
Sex	Male	42 (12%)
	Female	315 (88%)
Age, mean (SD)		44.8 (11.5)
Education	Basic training	48 (13%)
	Apprenticeship	82 (23%)
	College of higher education	90 (25%)
	University	75 (21%)
	Other	61 (17%)
Region	Southern Switzerland	223 (62%)
	North-eastern Switzerland	136 (38%)
Smoking	Yes	77 (21%)
	No	271 (76%)
	No Answer	9 (3%)
Pre-existing medical conditions	No	275 (77%)
	Yes	84 (23%)

As shown in Tables 2 and 86% of home health care workers reported work during hot days being rather or very strenuous. There was a statistically significant difference in reported perceived heat strain between home health care workers from north-eastern and southern Switzerland.

**Table 2:** Descriptive results for the outcome variable and independent variables of interest, by region

		Total	Region	
			North-east	South
Heat strain***	none / little	43 (12%)	27 (20%)	16 (7%)
	rather	151 (42%)	66 (49%)	85 (38%)
	very	159 (44%)	41 (30%)	118 (53%)
Workload change on hot days (> 30 °C)	Increased a lot	122 (34%)	34 (25%)	88 (40%)
	Increased somewhat	124 (35%)	68 (50%)	56 (25%)
	Same	97 (27%)	27 (20%)	70 (31%)
	Decreased somewhat	14 (4%)	7 (5%)	7 (3%)
Heat-related symptoms	No	21 (6%)	9 (7%)	12 (5%)
	Yes	324 (90%)	122 (90%)	202 (90%)
Individual adaptation measures	Avoid exertion	16 (5%)	9 (7%)	7 (3%)
	Adjust clothing***	195 (54%)	45 (33%)	150 (67%)
	Cool body	69 (19%)	22 (16%)	47 (21%)
	Seek cool environments***	35 (10%)	24 (18%)	11 (5%)
	Hydrate	186 (52%)	72 (53%)	114 (51%)
	Light eating	183 (51%)	69 (51%)	114 (51%)
	Nothing	55 (15%)	25 (18%)	30 (14%)
	Other measures	21 (6%)	9 (7%)	12 (5%)
	Reduce exertions***	45 (13%)	34 (25%)	11 (5%)
Transport by bicycle***		26 (7%)	21 (15%)	5 (2%)
Transport by e-Bike***		83 (23%)	76 (56%)	7 (3%)
Transport by car***		285 (79%)	72 (53%)	213 (96%)
Public transport		2 (0.6%)	1 (0.7%)	1 (0.4%)
Transport by other		7 (2%)	1 (0.7%)	6 (3%)
Break in cool area***	Yes	117 (33%)	62 (46%)	55 (25%)
	No	88 (25%)	36 (27%)	52 (23%)
	Sometimes	152 (42%)	38 (28%)	114 (51%)
Constrained hydration	Yes	155 (43%)	59 (43%)	96 (43%)
	No	123 (34%)	49 (36%)	74 (33%)
	Sometimes	79 (22%)	28 (21%)	51 (23%)

Statistical significance levels *p < 0.05, **p < 0.01, ***p < 0.001

Workload increasing a lot on hot days was reported by 34% of respondents, another 34% reported it increased somewhat. Whereas 96% in southern Switzerland reported traveling by car between clients, in north-eastern Switzerland 56% reported using an e-Bike and 15% reported using a bicycle–the differences being statistically significant. 43% of respondents reported frequently constraining their water intake, due to lack of time, or restroom availability and so forth. Here differences between regions were not statistically significant.

**Fig. 1:**
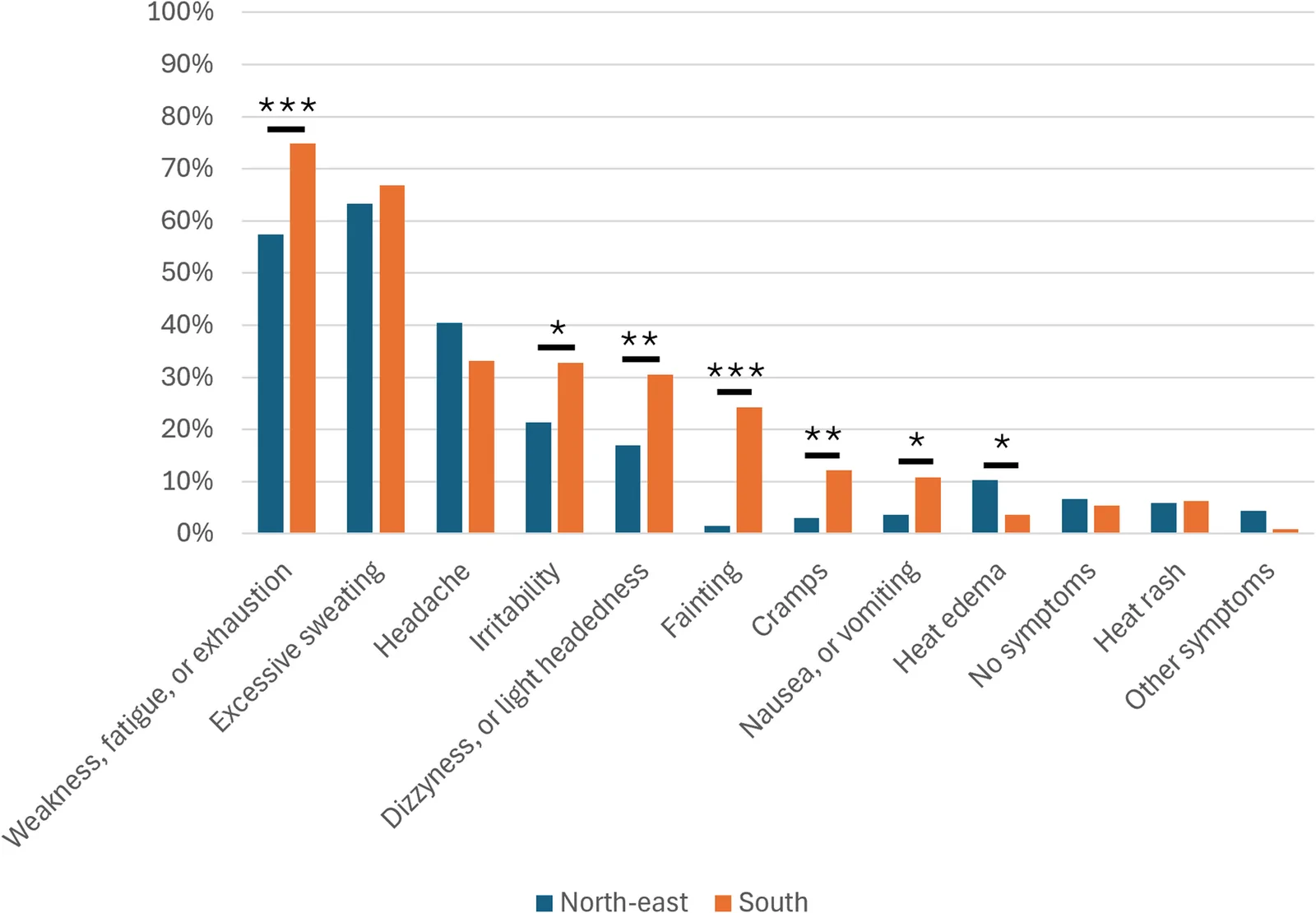
Individual heat-related symptoms (in percentage), by region. Note: Statistical significance levels *p < 0.05, **p < 0.01, ***p < 0.001

The majority of participants (90%) reported some symptoms that they attributed to heat exposure. The most frequently experienced symptoms were “weakness, fatigue, or exhaustion” (68%) and “excessive sweating” (65%) (Fig. 1). Rather frequent were also “headache” (36%), “irritability” (28%), and “dizziness, or light-headedness” (25%). There were significant differences between regions with workers in southern Switzerland experiencing higher frequences of symptoms than in north-eastern Switzerland.

### Factors associated with perceived heat strain

Table 3 reports the results from an ordinal logistic regression model for factors associated with perceived heat strain. A strong positive association was observed for reporting workload having increased a lot on hot days (OR 14.06 [7.12;28.85]) and for experiencing any heat-related symptoms (OR 5.27 [2.2; 12.88]). Working in southern Switzerland was associated with significantly higher heat strain (OR 3.98 [2.11; 7.61]). Use of the heat adaptation measure seeking cool environments was also significantly associated with higher perceived heat strain (OR 3.78 [1.55; 9.60]), as was having constrained hydration on hot days (OR 2.59 [1.35; 5.02]).

**Table 3:** Multivariate logistic regression model for factors associated with higher perceived heat strain in Switzerland

Variable	Level	OR	CI
Sex	Male	REF	
	Female	1.52	[0.7; 3.28]
Age		1	[0.97; 1.02]
Region	North-east	REF	
	South	**3.98**	**[2.11; 7.61]**
Smoking	Yes	1.29	[0.71; 2.36]
	No	REF	
	Prefer not to answer	5.63	[1; 38.9]
Education	Basic training	1.25	[0.54; 2.9]
	Apprenticeship	REF	
	College of higher education	0.93	[0.46; 1.86]
	University	1.01	[0.47; 2.17]
	Other	1.06	[0.47; 2.37]
Pre-existing medical condition	Yes	1.13	[0.63; 2.05]
	No	REF	
Workload change on hot days (> 30 °C)	Increased a lot	**14.06**	**[7.12; 28.85]**
	Increased somewhat	1.48	[0.84; 2.65]
	Same	REF	
	Decreased somewhat	0.79	[0.24; 2.63]
Heat-related symptoms	Yes	**5.27**	**[2.2; 12.88]**
	No	REF	
Individual adaptation measures	Avoid exertion	0.54	[0.17; 1.71]
	Adjust clothing	0.79	[0.44; 1.39]
	Cool body	1.63	[0.82; 3.29]
	Seeking cool environments	**3.78**	**[1.55; 9.6]**
	Hydrate	1.32	[0.73; 2.42]
	Light eating	0.79	[0.47; 1.32]
	Nothing	0.64	[0.28; 1.47]
	Other measures	0.73	[0.27; 2.04]
	Reduce exertions	0.98	[0.47; 2.08]
Break in cool area possible	Yes	0.58	[0.3; 1.14]
	No	REF	
	Sometimes	0.69	[0.36; 1.31]
Constrained hydration	Yes	**2.59**	**[1.35; 5.02]**
	No	REF	
	Sometimes	1.3	[0.66; 2.56]

Statistically significant (*p* < 0.05) results are highlighted in bold

*OR* Odds ratios, *CI* 95% confidence intervals

## Discussion

This study examines the heat strain experienced among 359 home health care workers during the summer of 2024 in two regions in Switzerland and provides novel data on heat strain in this occupational group. Our descriptive analysis showed that 86% of home health care workers perceived work to be rather or very strenuous on hot days. About the same share of workers, 90%, reported heat-related symptoms. By far the most frequent symptoms that home health care workers experienced on hot days were “weakness, fatigue, or exhaustion” and “excessive sweating”. As pointed out by Rossi et al. [8] in their systematic review, two other studies which addressed heat-related symptoms also found high prevalence of these symptoms, namely Venugopal et al. [36], and Suter et al. [37] and our results are thus consistent with previous research. While both of these studies were conducted in tropical climates (India and Indonesia), our study offers evidence for a temperate climate.

As expected, we found regional differences between southern and north-eastern Switzerland regarding perceived heat strain, with workers in southern Switzerland experiencing greater perceived heat strain than those in north-eastern Switzerland. This is plausible given the average temperature differences between these two regions [24]. 

Moreover, our ordinal logistic regression analysis provided two novel insights. First, perceived heat strain was associated with a strong increase in workload on hot days. This may reflect the physiological burden of physical work, which elevates endogenous heat production and complicates thermal regulation, as established by prior literature [38]. 

Second, there was a relationship between perceived heat strain and workers’ heat-related behaviour. Participants who reported constraining their water intake also reported higher perceived heat strain. It is plausible that constrained hydration is associated with higher perceived heat strain. This assumption is supported by previous research showing that sufficient hydration positively affects heat tolerance [39]. 

Amongst individual adaptation measures, perceived heat strain was significantly associated with seeking cool environments. This suggests that home health care workers perceiving heat as more strenuous report a corresponding increase in seeking cool environments [28]. 

### Strengths and limitations

Strengths of our study are, first, that it is the first study on heat strain among home health care workers; second, the inclusion of two regions of Switzerland that differ substantially in terms of climate; and third, that workers of all working age groups participated.

Limitations of our study are, first, that participants were recruited using convenience and snowball sampling. This approach likely leads to an overrepresentation of workers experiencing significant heat strain, as those with milder symptoms or higher thermal tolerance may have been less inclined to participate. Consequently, reported prevalence rates may overestimate the population burden. Future studies should employ representative sampling or prospective monitoring to establish generalizable estimates. Second, heat exposure, physiological strain, and hydration status were assessed exclusively through self-report, without concurrent objective measurements of environmental heat exposure or physiological indicators. Consequently, both outcome and independent variables are subjective and therefore susceptible to recall and reporting bias, which is an inherent limitation of cross-sectional survey studies. Third, to keep the survey as brief as possible and to focus on factors addressable in a heat adaptation strategy in this context, we did not collect data on some potential covariates (e.g. BMI, menopausal status). Fourth, the study is context-bound to the Swiss home health care system; consequently, the transferability of these estimates to other contexts—particularly those with different climates or regulatory frameworks—remains limited. In accordance with the sex distribution of home health care workers, the sample is mostly comprised of women. Therefore, our study provides good data for women, but it cannot investigate gender differences or specific features of heat strain among men.

### Implications and further research directions

Our findings indicate that heat exposure seems to put substantial strain on the home health care workers in our sample. Thus, protective and supportive measures tailored to workplace characteristics—are warranted such as workload, constrained hydration, and geographical region—to strengthen climate change resilience in this important and growing economic sector. Given home health care workers’ particular working conditions—which do not allow them or their employers to regulate the room temperature in which they work—they require tailored measures to address the challenge of heat strain on hot days. For instance, measures to reduce workload on hot days, or to improve hydration seem warranted.

Given that home health care workers in our sample experience substantial heat strain at work, future research directions may be the development of appropriate heat protection measures and related testing of such measures in a randomized controlled trial.

## Conclusion

Research on heat strain among home health care workers remains limited, even though this workforce is likely to be particularly exposed to heat due to a combination of outdoor commutes, physically demanding care tasks, and constrained influence on their working environment. This study contributes new empirical evidence by providing a detailed assessment of perceived heat strain and its associated factors among home health care workers in Switzerland.

## Supplementary Information

Supplementary Material 1.

## Data Availability

In accordance with SNSF requirements, the full data set will be published and can be accessed under the following DOI (https://doi.org/10.48573/nrhm-sg66). The used questionnaire is also accessible there.

## Abbreviations

PPE: Personal protective equipment
