# Supplementary material for: Occupational heat strain among home health care workers: a cross-sectional study
Source: BMC Public Health. 2026 Sep 29;26:2690. doi: 10.1186/s12889-026-28969-8 (PMC13621764; doi:10.1186/s12889-026-28969-8)
Supplement: Supplementary file 1 [file 12889_2026_28969_MOESM1_ESM.pdf]

Heat Survey for Home Care Workers

REHEAT – Heat Resilience and Health in Home Care Services

Introduction

Hello,

Thank you very much for participating in this survey.

As part of the four-year REHEAT project, we are investigating the effects of heatwaves on the well-being and effectiveness of heat protection measures among home care health professionals. This questionnaire aims to assess your level of heat-related strain during work. We also want to learn about the heat protection measures you currently use and which ones you would like to see implemented in future heatwaves. Your responses will help us develop effective heat protection strategies for the workplace, which can be tested within the framework of the REHEAT project.

The project is being conducted by the Department of Health Sciences at ZHAW (Zurich University of Applied Sciences), in collaboration with SUPSI (University of Applied Sciences and Arts of Southern Switzerland) and SwissTPH (Swiss Tropical and Public Health Institute).

This survey is completely anonymous. The REHEAT research team, which will analyze the data, has no access to names or addresses. After the project concludes, the data will be anonymized and published to allow reuse in future studies.

Participation is voluntary, and you may withdraw at any time. The survey takes approximately 5 minutes to complete.

For more information, please visit:
https://www.zhaw.ch/de/forschung/forschungsdatenbank/projektdetail/projektid/7273/

For questions, please contact the co-project leader:
Prof. Dr. Isabel Baumann
Email: isabel.baumann@zhaw.ch

If you agree to the anonymous collection, storage, and analysis of your data, please continue.
If you do not consent, you may end the survey at any time.

Demographic Information

What is your sex?

- ☐ Male
- ☐ Female
- ☐ Prefer not to say

How old are you?

……………… (years)

What is your highest completed education?

- ☐ Health Care Assistant (Swiss Red Cross)
- ☐ Healthcare and Social Care Assistant (Federal Certificate of Vocational Education and Training)
- ☐ Specialist in Care (Federal Diploma of Vocational Education and Training)
- ☐ Healthcare Specialist (Federal Diploma of Vocational Education and Training)
- ☐ College of Higher Education in Nursing (Advanced Federal Diploma of Higher Education)
- ☐ Bachelor's degree (BSc or BA) from a University of Applied Sciences or University
- ☐ Master's degree (MSc or MA) from a University of Applied Sciences or University
- ☐ Doctorate
- ☐ Other: ……………

What is your current work percentage in home care services (0–100%)?

………….. (%)

How long have you been working in your occupation?

- ☐ Less than 1 year
- ☐ 1–2 years
- ☐ 3–4 years
- ☐ 5–9 years

- ☐ 10–14 years
- ☐ 15+ years

Work and Transportation

Which mode of transport do you use when traveling between clients?

- ☐ Bicycle
- ☐ Electric bicycle (e-bike)
- ☐ Public transportation
- ☐ Other: …………..

Heat Exposure and Health Impact

How strenuous is the heat for you at work on hot days (>30°C)??

- ☐ Very strenuous
- ☐ Rather strenuous
- ☐ A little strenuous
- ☐ Not at all

On very hot days (>30°C), do you experience any of the following symptoms while working?

(Check all that apply)

- ☐ Dizziness or light headedness
- ☐ Fainting
- ☐ Weakness, fatigue, or exhaustion
- ☐ Headache
- ☐ Nausea or vomiting
- ☐ Cramps (especially in calves and legs)
- ☐ Irritability
- ☐ Heat rash
- ☐ Heat edema (swelling due to heat)

- ☐ Excessive sweating
- ☐ Other symptoms: ……………..
- ☐ No symptoms

Compared to a typical workday, how has your workload changed on very hot days (>30°C)?

- ☐ Increased a lot
- ☐ Increased somewhat
- ☐ Same
- ☐ Decreased somewhat
- ☐ Decreased a lot

Heat Protection Measures

Does your organization/employer provide recommendations on how to deal with extreme heat (>30°C) at work?

- ☐ Yes
- ☐ No
- ☐ I don’t know

On very hot days (>30°C), can you take breaks in a cool place?

- ☐ Yes
- ☐ No
- ☐ Sometimes

On very hot days (>30°C), do you drink less than you would like (e.g., due to time constraints or to avoid frequent toilet use)?

- ☐ Yes
- ☐ No
- ☐ Sometimes

Personal Heat Protection Measures

What did you do at work this summer to protect your own health from the heat? *(Check all that apply)*

- ☐ Nothing, no concrete action taken
- ☐ Avoid exertion; postpone strenuous work until cooler times of the day
- ☐ Reduce exertion; reduce work pace during strenuous activities
- ☐ Avoid warm/hot rooms; seek out cool places
- ☐ Adjust clothing: Wear light/airy clothes
- ☐ Cool body: e.g., with cool showers, cold cloths on forehead or neck, cold foot and hand baths
- ☐ Drink enough
- ☐ Adjust diet: eat light meals, smaller portions, more refreshing foods
- ☐ Other strategies: ………….

Suggestions for Future Heat Protection

Do you have any suggestions or wishes for heat protection measures that would help you during periods of high heat?

……………………………………………………………………………………………………..

Health Conditions

Do you suffer from any of the following conditions?

(Check all that apply)

- ☐ Heart or circulatory system disorders (e.g., high blood pressure, heart failure, coronary heart disease, arrhythmias, heart attack)
- ☐ Lung or respiratory tract diseases (e.g., asthma, chronic bronchitis, COPD, recurrent pneumonia, pulmonary fibrosis)
- ☐ Diabetes / sugar disease
- ☐ Immune deficiency or cancer (immune system problems or immune suppression due to treatment such as chemotherapy or transplantation)
- ☐ Kidney disease (especially chronic reduction in kidney function due to high blood pressure, diabetes, inflammation, autoimmune conditions, or congenital defects)

- ☐ Mental health condition (e.g., depression, mania, schizophrenia, psychosis, anxiety disorders, post-traumatic stress disorder)
- ☐ Other chronic illnesses such as neurological disorders or gastrointestinal diseases
- ☐ No
- ☐ Prefer not to say

Lifestyle

Do you smoke?

- ☐ Yes
- ☐ No
- ☐ Prefer not to say

Thank You

Thank you again for your participation. Your input is valuable in helping us improve working conditions during heatwaves.

Project: REHEAT – Heat Resilience and Health in Home Care Services
Conducted by ZHAW, SUPSI, and SwissTPH
Data will be anonymized and used for research purposes only
